# Supplementary material for: Cell Type-Specific Survey of Epigenetic Modifications by Tandem Chromatin Immunoprecipitation Sequencing
Source: Sci Rep. 2018 Jan 18;8:1143. doi: 10.1038/s41598-018-19494-9 (PMC5773701; doi:10.1038/s41598-018-19494-9)
Supplement: Supplementary file 1 — Supplemental materials [file 41598_2018_19494_MOESM1_ESM.pdf]

Cell Type-Specific Survey of Epigenetic Modifications by Tandem Chromatin Immunoprecipitation Sequencing

Mari Mito, Mitsutaka Kadota, Kaori Tanaka, Yasuhide Furuta, Kuniya Abe, Shintaro Iwasaki, and Shinichi Nakagawa

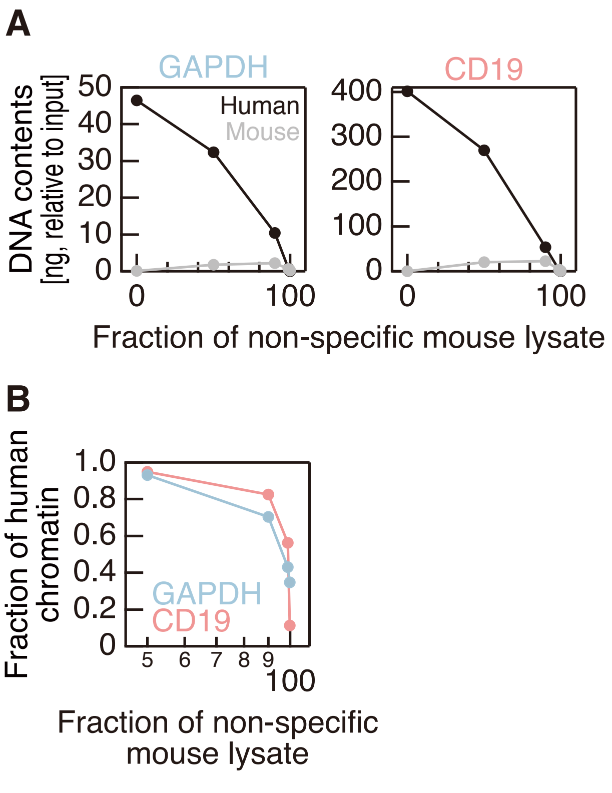

Supplemental Figure S1 Mito *et al.* (2017)

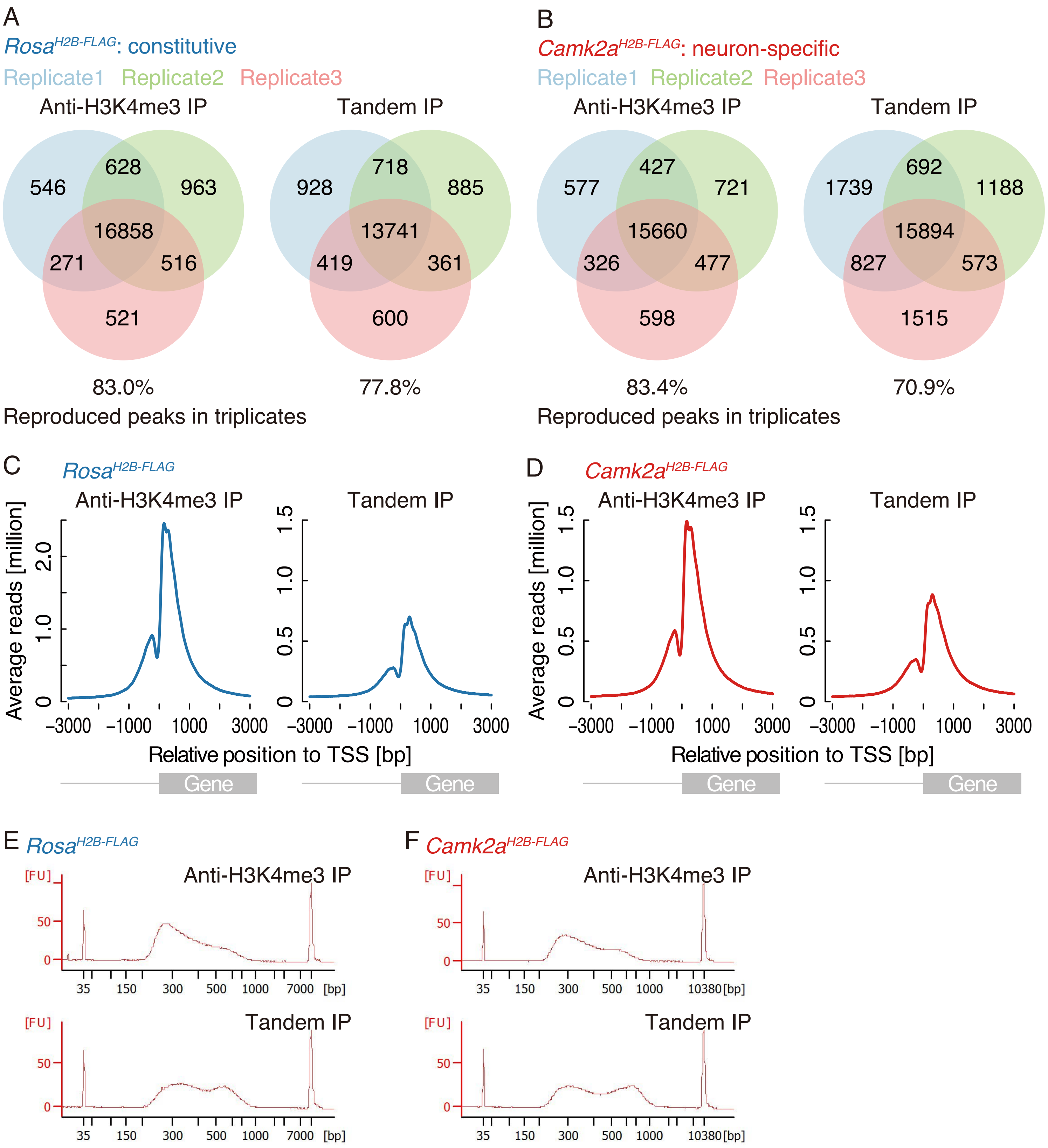

Supplemental Figure S2 Mito *et al.* (2017)

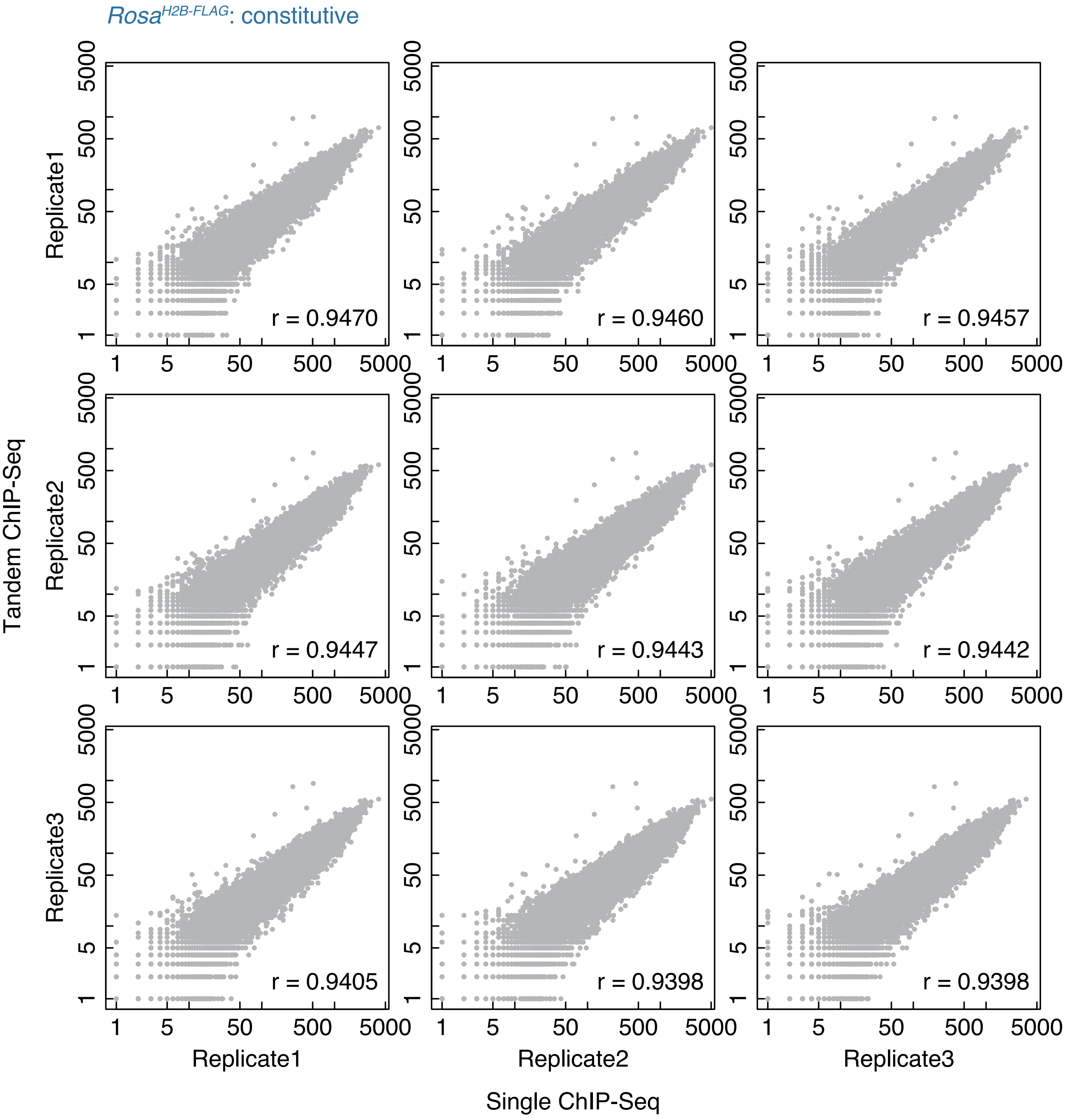

Supplemental Figure S3 Mito *et al.* (2017)

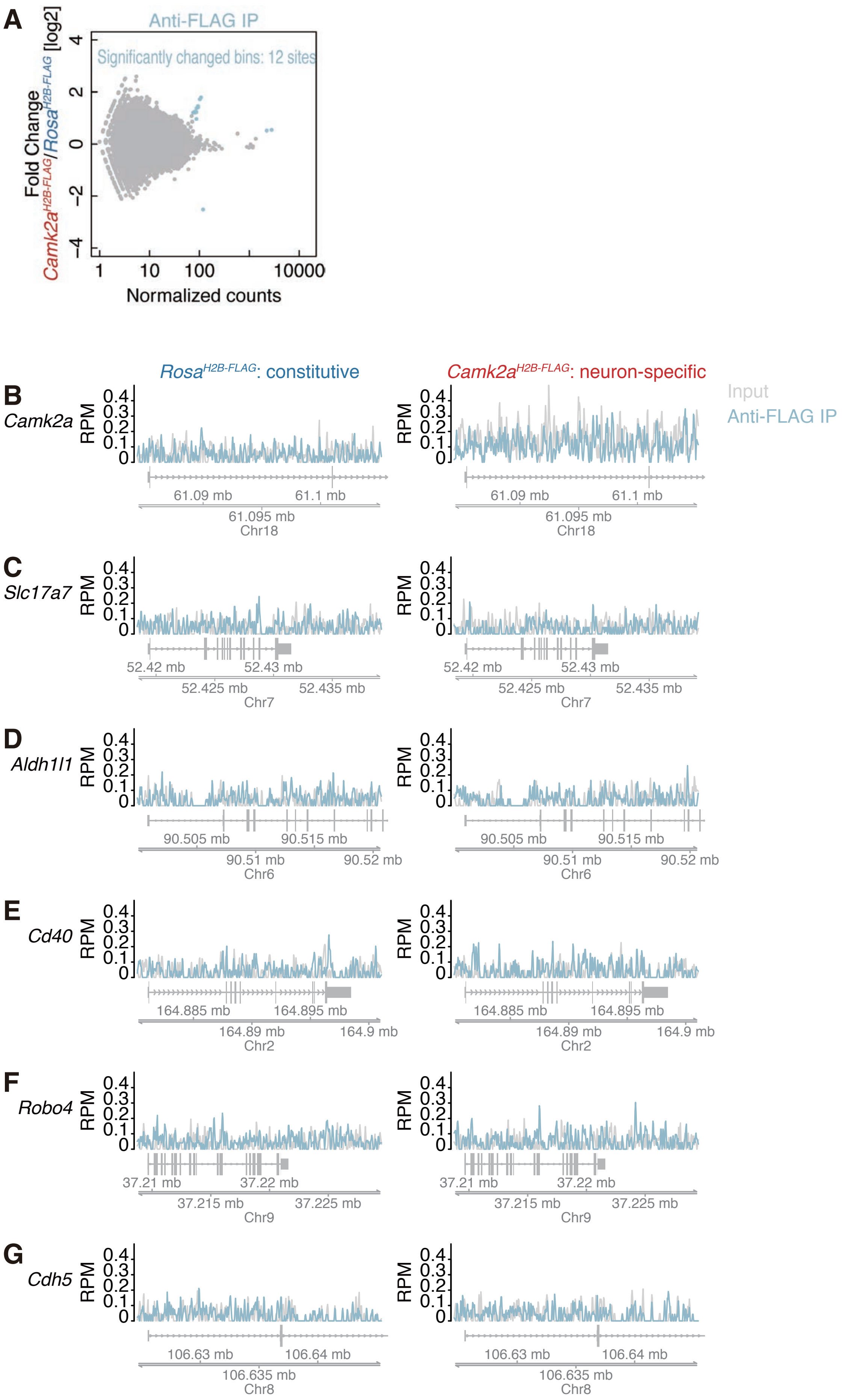
A
Anti-FLAG IP
Significantly changed bins: 12 sites
Fold Change Camk2a^H2B-FLAG/Rosa^H2B-FLAG [log2]
Normalized counts
B
Camk2a
C
Slc17a7
D
Aldh1l1
E
Cd40
F
Robo4
G
Cdh5
Rosa^H2B-FLAG: constitutive
Camk2a^H2B-FLAG: neuron-specific
Input
Anti-FLAG IP
RPM
Chr18
Chr7
Chr6
Chr2
Chr9
Chr8

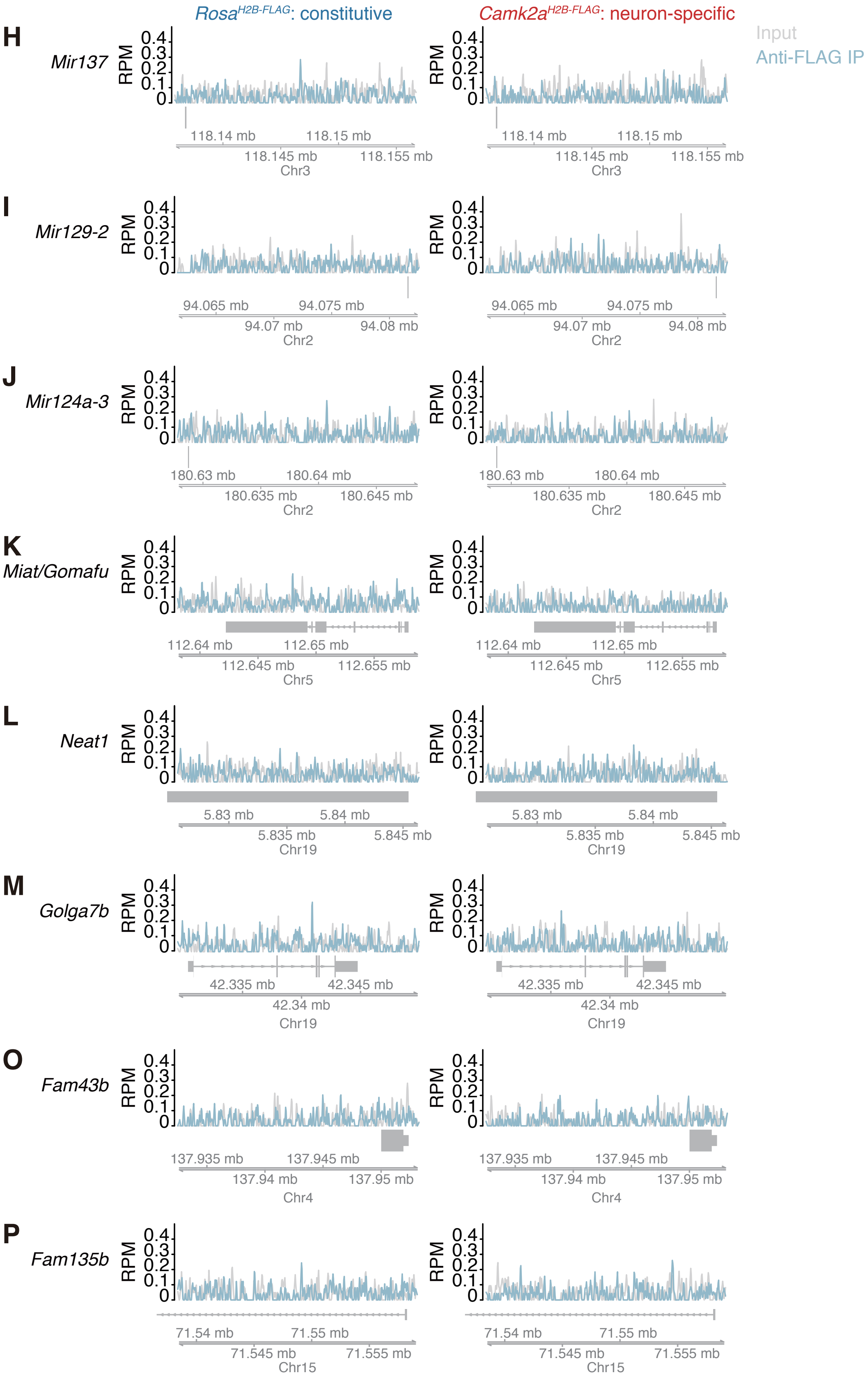

Supplemental Figure S4 Mito *et al.* (2017)

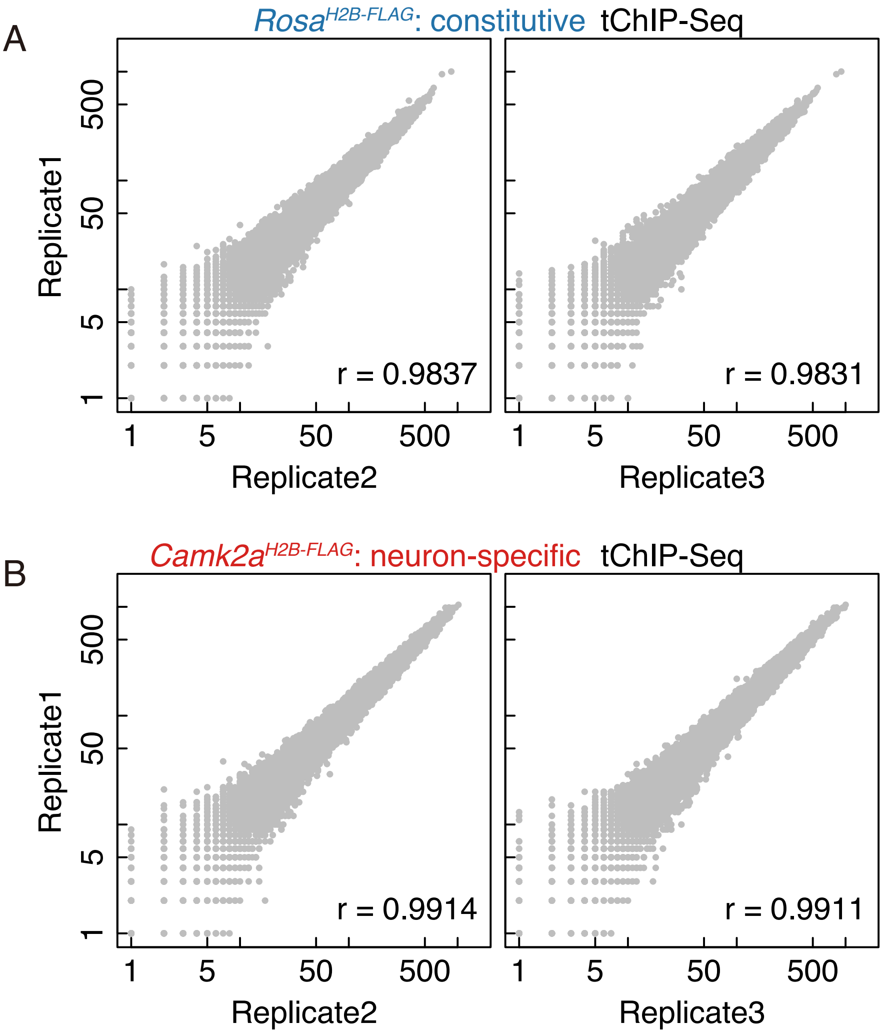

Supplemental Figure S5 Mito *et al.* (2017)

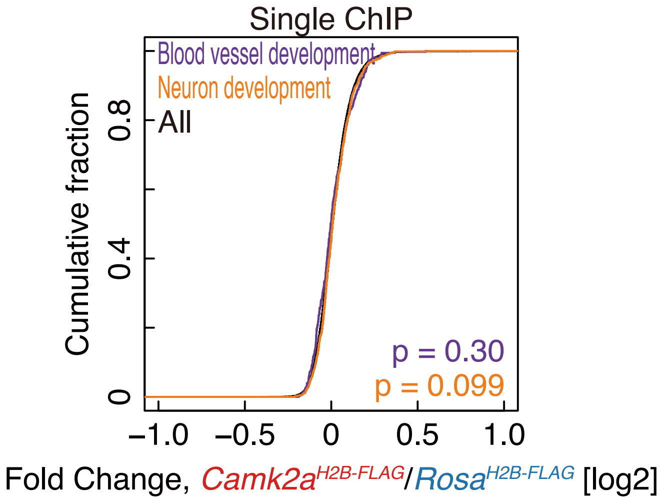

Supplemental Figure S6 Mito *et al.* (2017)

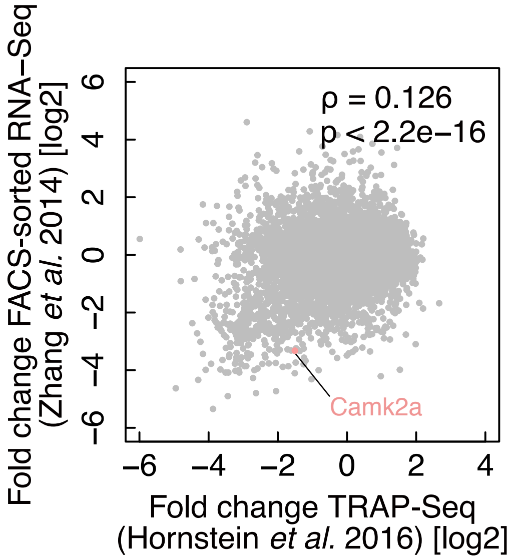

Supplemental Figure S7 Mito *et al.* (2017)

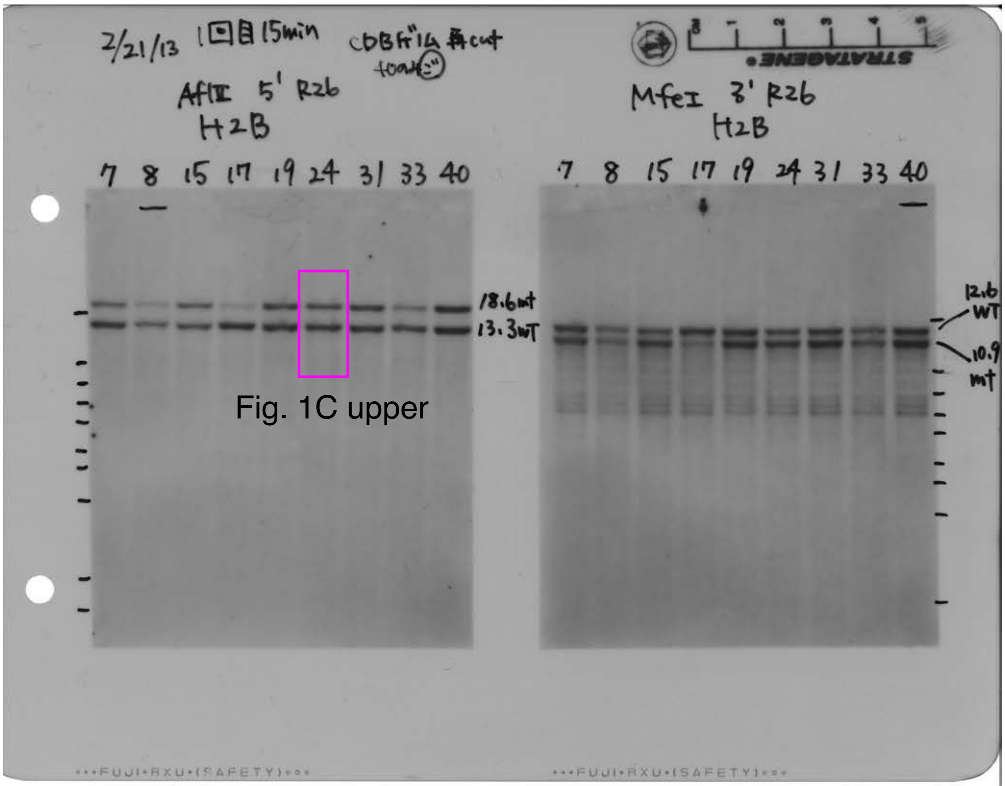

Fig. 1C upper

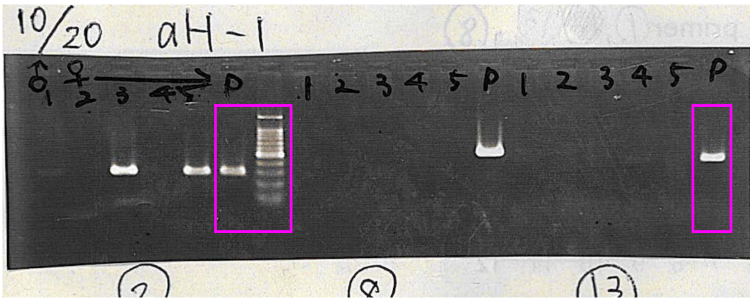

Fig. 1C lower

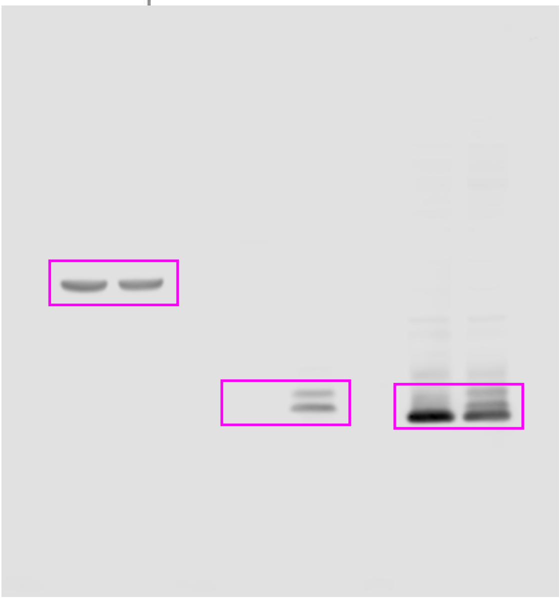

Fig. 1E

Supplemental figure legends

Supplemental figure S1. Limitation of target cell population for tChIP-Seq analysis.

(A and B) DNA bound to H2B-FLAG quantified by *GAPDH* and *CD19* qPCR. Lysate of HEK293 cells expressing H2B-FLAG and that of non-specific NIH3T3 cells were mixed at different ratios prior to FLAG purification.

(B) Ratio between specific human fraction and contaminated mouse fraction was shown.

Supplemental figure S2. Characterization of peaks called in tChIP-Seqs from *Rosa*$^{H2B\text{-}FLAG}$ and *Camk2a*$^{H2B\text{-}FLAG}$.

(A-B) Overlap of peaks called in triplicates of H3K4me3 single and tandem ChIP-Seqs.

(C-D) Enrichments of reads along the transcription start site (shown at 0 on the x-axis). TSS: transcription start site.

(E) Library sizes for single and tandem of ChIP-Seqs. H2B-FLAG purification tends to isolate a population of longer DNA fragments.

Supplemental figure S3. Correlations of reads mapped to H3K4me3 peaks in single and tChIP-Seqs from *Rosa*$^{H2B\text{-}FLAG}$

r is Pearson's correlation coefficient.

Supplemental figure S4. Unbiased access to cell-type specific chromatin.

(A) MA-plot of the mean reads in every 10-kbp bin along the entire genome versus their differential changes in anti-FLAG IP between *Rosa*$^{H2B\text{-}FLAG}$ and *Camk2a*$^{H2B\text{-}FLAG}$. Significantly changed bins (FDR < 0.01) are highlighted in light blue.

(B-P) Distribution of reads of input and anti-FLAG IP obtained from *Rosa*$^{H2B\text{-}FLAG}$ and *Camk2a*$^{H2B\text{-}FLAG}$ mice along genes analyzed in this study. RPM: reads per million.

Supplemental figure S5. Correlations of reads mapped to H3K4me3 peaks in tChIP-Seqs from *Rosa*$^{H2B\text{-}FLAG}$ (A) and *Camk2a*$^{H2B\text{-}FLAG}$ (B).

r is Pearson's correlation coefficient.

Supplemental figure S6. Differential changes observed between single tChIP-Seqs from *Rosa*$^{H2B\text{-}FLAG}$ and *Camk2a*$^{H2B\text{-}FLAG}$.

Same as Fig. 3E, but for single H3K4me3 ChIP-Seq.

Supplemental figure S7. Comparison of previously reported methods for neuron-specific gene expression.

Correlation of TRAP-seq enrichment and FACS-sorted RNA-Seq. ρ : Spearman's rank correlation. P value is calculated by Student's t test. Camk2a is indicated in pink color.

Supplemental figure S8. Original gel/blot images for figure 1.

Supplemental table T1. The list of neuronal promoters identified by tChIP-Seq.

Each locus of the H3K4me3 peaks is listed with genomic position, distance to transcription start site (TSS), reads fold change in tChIP-Seq $Camk2a^{H2B\text{-}FLAG}$ compared to that of $Rosa^{H2B\text{-}FLAG}$, q value, assigned gene name, gene description, Ensembl id, and RefSeq IDs.
